# Evaluating the potential of quantitative assessment of intraoperative fasciocutaneous flap perfusion using microscope-integrated indocyanine green fluorescence angiography

**DOI:** 10.1016/j.jpra.2026.01.008

**Published:** 2026-01-16

**Authors:** Lasse W.P. van ’t Hof, David J. Nijssen, Richard M. van den Elzen, Joost R. van der Vorst, Roel Hompes, Mark-Bram Bouman, Mark I. van Berge Henegouwen, Matthijs Botman, Caroline Driessen

**Affiliations:** a Department of Plastic, Reconstructive and Hand surgery, Amsterdam UMC, Amsterdam, the Netherlands; b Amsterdam Movement Sciences, Amsterdam UMC, Amsterdam, the Netherlands; c Department of Surgery, Amsterdam UMC, University of Amsterdam, Amsterdam, the Netherlands; d Cancer Center Amsterdam, Imaging and Biomarkers, Amsterdam, the Netherlands; e Amsterdam UMC Location University of Amsterdam, Biomedical Engineering and Physics, Amsterdam, the Netherlands; f Department of Surgery, Leiden University Medical Center, Leiden, the Netherlands

**Keywords:** Indocyanine green (ICG), Fluorescence angiography (FA), Quantitative ICG-FA, Perfusion assessment, Reconstructive surgery

## Abstract

**Background:**

Perfusion-related complications are a leading cause of morbidity and flap loss in reconstructive surgery. While indocyanine green fluorescence angiography (ICG-FA) is commonly used, its subjective interpretation may miss early signs of perfusion disturbances. Quantitative ICG-FA (Q-ICG-FA) offers a more objective method, but its potential in fasciocutaneous flaps remains insufficiently studied.

**Methods:**

This retrospective study analyzed intraoperative ICG-FA data from patients undergoing fasciocutaneous flap reconstruction. Fluorescence-time curves (FTCs) were generated from three regions of interest (ROIs): reference tissue (ROI1), proximal flap (ROI2), and distal flap (ROI3). FTCs and derived quantitative parameters, including time-to-peak (Ttp) and normalized inflow/outflow slopes, were compared between patients with (PRC) and without (No PRC) perfusion-related complications.

**Results:**

Twenty patients were included. Seven patients experienced a perfusion-related complication, most commonly venous congestion (*n* = 5). FTCs of compromised flaps showed delayed fluorescence peaks and reduced washout, most pronounced in ROI3. Quantitatively, the PRC group showed prolonged Ttp in ROI2 (105 vs. 36 s, *p* = 0.013) and ROI3 (209 vs. 48 s, *p* = 0.001), lower normalized mean inflow slopes in ROI2 (2.4 vs 0.9%/s, *p* = 0.029) and ROI3 (4.2 vs. 1.6%/s, *p* = 0.002), and reduced mean outflow slopes in ROI3 (0.1 vs. 0.2, *p* = 0.021). Conventional clinical assessments failed to identify all compromised flaps.

**Conclusion:**

Q-ICG-FA objectively distinguished flaps with and without perfusion-related complications. Dynamic parameters, particularly Ttp and inflow/outflow slopes, were most associated with perfusion compromise. Larger studies are needed to validate these findings.

## Introduction

Fasciocutaneous flaps are widely used in reconstructive surgery because of their reliability, versatility and minimal donor site morbidity.^1^^,^^2^ These flaps consist of skin, subcutaneous tissue, and underlying fascia and may be transferred as pedicled or free flaps.^3^ Their anatomical composition enables the transfer of well-vascularized soft tissue, supported by vascular patterns that may be random, axial, or perforator based.^4^^,^^5^ The wide range of available donor sites allows surgeons to tailor flap selection to specific reconstructive needs. Despite their widespread clinical use, flap failure remains a relevant risk. Ensuring adequate vascularization is crucial to prevent perfusion-related complications, which may arise from arterial, venous, or microcirculatory insufficiency and can lead to necrosis or infection.^6^, ^7^, ^8^ The likelihood of such complications may vary depending on the flap transfer type and underlying vascular pattern.^9^^,^^10^

Intraoperative assessment of flap viability is traditionally based on clinical indicators such as capillary refill, temperature, color, and bleeding at the wound edges.^11^ Over the past decade, indocyanine green fluorescence angiography (ICG-FA) has become a valuable imaging tool for real-time intraoperative perfusion assessment.^12^^,^^13^ After intravenous injection, indocyanine green (ICG) binds to plasma proteins and remains intravascular; when exposed to near-infrared light, it emits fluorescence that allows visualization of tissue perfusion.^14^ In reconstructive surgery, ICG-FA is well established in breast reconstruction, where it supports intraoperative identification of mal-perfused tissue.^15^, ^16^, ^17^ The integration of near-infrared fluorescence modules into modern surgical microscopes has made ICG-FA increasingly accessible, particularly in microsurgical procedures.^18^^,^^19^

Conventional interpretation of ICG-FA relies on the surgeon’s subjective evaluation of the fluorescence pattern. However, this approach is prone to interobserver variability and may fail to detect early or subtle perfusion abnormalities.^20^^,^^21^ Objective analysis of fluorescence dynamics can reveal subtle disturbances that are not apparent on visual inspection.^22^ Quantitative ICG-FA (Q-ICG-FA) enables such analysis by converting fluorescence intensity into numerical values.^23^ Plotting these values over time generates a fluorescence–time curve (FTC), from which multiple perfusion parameters, such as inflow slope, time-to-peak, and wash-out characteristics, can be derived.^24^, ^25^, ^26^

The aim of this pilot study was to evaluate whether microscope-integrated Q-ICG-FA can distinguish patients with and without perfusion-related complications in fasciocutaneous flap reconstruction. Assessing perfusion dynamics through FTC patterns may provide insights into flap perfusion behavior and help identify early signs of tissue compromise.

## Materials and methods

### Study design and setting

This pilot study is a retrospective analysis of prospectively collected data from an ongoing single-center clinical study at Amsterdam UMC (ClinicalTrials.gov: NCT06046982). Intraoperative ICG-FA recordings and postoperative follow-up assessments were obtained according to a standardized protocol. Patients were enrolled between August 2023 and October 2024. The study protocol was approved by the Institutional Review Board (IRB) of Amsterdam UMC (NL74852.029.21). This article is reported in accordance with STROBE guidelines.^27^

### Participants

The study included consecutive patients aged ≥18 years undergoing reconstructive surgery with a fasciocutaneous flap at any anatomical site. Potential study participants were identified from the surgical planning list and during the multidisciplinary team meetings (MDT). Exclusion criteria were: known allergies to ICG, iodide, or shellfish; hyperthyroidism; pregnancy or lactation; epilepsy; and severe hepatic or renal failure. Patients were also excluded if postoperative flap inspection was not possible or if ICG-FA was not recorded using the Zeiss microscope-integrated system. Postoperative follow-up consisted of clinical evaluations on postoperative days 1, 2, and 3, and at approximately 2 and 6 weeks in the outpatient clinic.

### Standardized ICG-FA assessment

**Figure 1:**
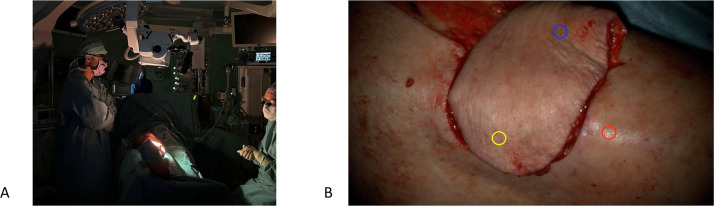
(a) Intraoperative setup of the Zeiss Tivato 700 microscope equipped with a built-in near-infrared camera. (b) Field of view at a camera distance of 60 cm with a magnification of 1,6x. The red circle marks the reference tissue outside the flap (ROI1), the blue circle marks the proximal area of the flap (ROI2) and the yellow circle marks the distal area of the flap (ROI3). ROI, region of interest.

ICG-FA was performed after flap transfer or following completion of the vascular anastomosis using a Zeiss Tivato 700 microscope equipped with the INFRARED 800 module (excitation 700–780 nm; emission 820–900 nm). The microscope was positioned perpendicularly at a fixed distance of 60 cm (Figure 1A) to simultaneously visualize the flap and reference tissue (Figure 1B). Settings were standardized: 1.6 × magnification, 100% light intensity, and fixed gain. Overhead lights were switched off, and a white-light image was obtained for orientation. ICG (Verdye) was administered intravenously at 0.1 mg/kg followed by a 10 mL saline flush. Fluorescence was recorded continuously for 5 min. Hemodynamic parameters (heart rate, systolic/diastolic blood pressure, and MAP) were documented during imaging. As this was a pilot study, intraoperative management was not influenced by ICG-FA findings. During the study period, multiple ICG imaging modalities were used; only cases imaged with the Zeiss INFRARED 800 system were eligible for this study.

### Quantification of ICG-FA

The quantification methodology has been described previously.^18^^,^^28^^,^^29^ Postoperative analysis was performed using custom software developed at Amsterdam UMC in Python v3.8 (Python Software Foundation, https://www.python.org/). After selecting regions of interest (ROIs), the software automatically generated FTCs and calculated the corresponding quantitative parameters.

**Figure 2:**
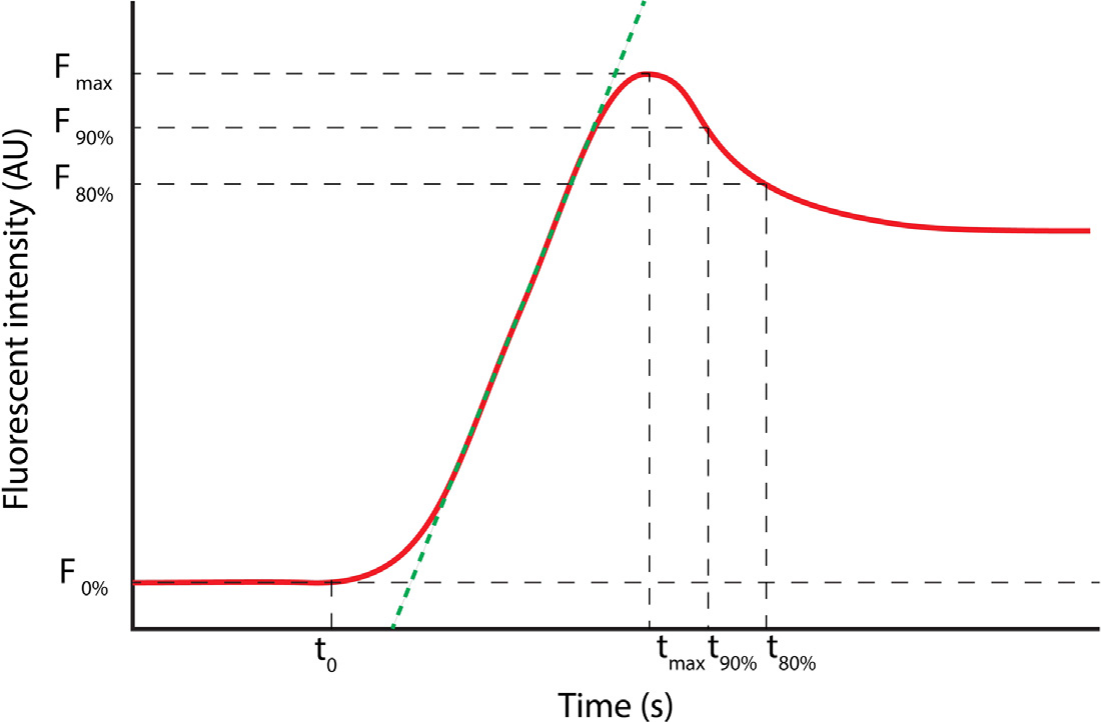
Fluorescence-time curve (FTC) of ICG angiography for quantitative perfusion analysis containing the following parameters: The inflow time point (t0) marks the specific moment when the fluorescence intensity within the region of interest (ROI) first exceeds the background level significantly. Fmax represents the maximum fluorescence intensity, measured in arbitrary units (AU), and Tmax is the time at which this background-corrected fluorescence intensity reaches Fmax. The time-to-peak (Ttp) is calculated as the difference between Tmax and t0. The green line on the curve represents the mean slope, which indicates the rate of fluorescence intensity increase, measured in AU/s. Please note that this figure has been previously made by our group and has been published in Surgical Endoscopy by J.J. Joosten et al. (https://doi.org/10.1007/s00464-023-10107-9) (color figure online).

Three ROIs were defined for each case: ROI1 (red) was placed in a reference area outside the flap, ROI2 (blue) in flap tissue proximal to the supplying vessel, and ROI3 (yellow) in flap tissue distal to the supplying vessel (Figure 1B). This configuration enabled assessment of intra-flap perfusion gradients and comparison with reference tissue. A schematic representation of a FTC and its derived parameters is shown in Figure 2.

### Outcomes and definitions

The primary outcome was the FTC and the associated Q-ICG-FA parameters. FTCs and parameters were compared between patients with and without perfusion-related complications. The quantitative parameters included the time from t0 to maximum fluorescence intensity (*Ttp*), *the* *normalized* *mean* *slope* *inflow*, the *normalized* *maximum* *slope* *inflow*, the *normalized* *mean* *slope* *outflow*, and the *normalized* *maximum* *slope* *outflow*. Normalization of the curve was applied by plotting the fluorescence intensity over time as a percentage of the maximum fluorescence intensity ‘*Fmax*’, thereby reducing case-specific variability and enabling better comparison of FTCs across cases.^30^

Secondary outcomes included perfusion-related complications, reoperation rates, flap survival, intraoperative clinical assessment, and intraoperative hemodynamic parameters. Perfusion-related complications were identified during the in-hospital follow-up as well as at 2 and 6 weeks postoperatively, based on clinical signs of compromised tissue viability. These complications were classified as skin necrosis, venous congestion, and wound complications due to inadequate perfusion. Complications were graded according to the Clavien-Dindo classification,^31^ with only grade III or higher considered clinically significant. Patients were then categorized into two groups: the PRC group (patients with perfusion-related complications) and the No PRC group (patients without perfusion-related complications). Intraoperative clinical assessment of flap viability was based on conventional clinical indicators, including capillary refill, tissue color, temperature, and bleeding at the wound edges. Flaps were further categorized by transfer type (pedicled vs. free) and vascular pattern (random, axial, or perforator).

### Statistics

Baseline patient characteristics are summarized using descriptive statistics. Categorial data are presented as frequencies and percentages, whilst continuous data are shown as mean ± standard deviation or as median (range), depending on the data distribution. Categorial and continuous variables are compared using a chi-square test or Fisher’s exact, and the unpaired t-test or Mann-Whitney U test, respectively. A *p*-value <0.05 was considered statistically significant. Data was analyzed using the Statistical Package for Social Sciences (SPSS) of IBM Statistics, version 28.0.

## Results

### Patient demographics and baseline characteristics

**Figure 3:**
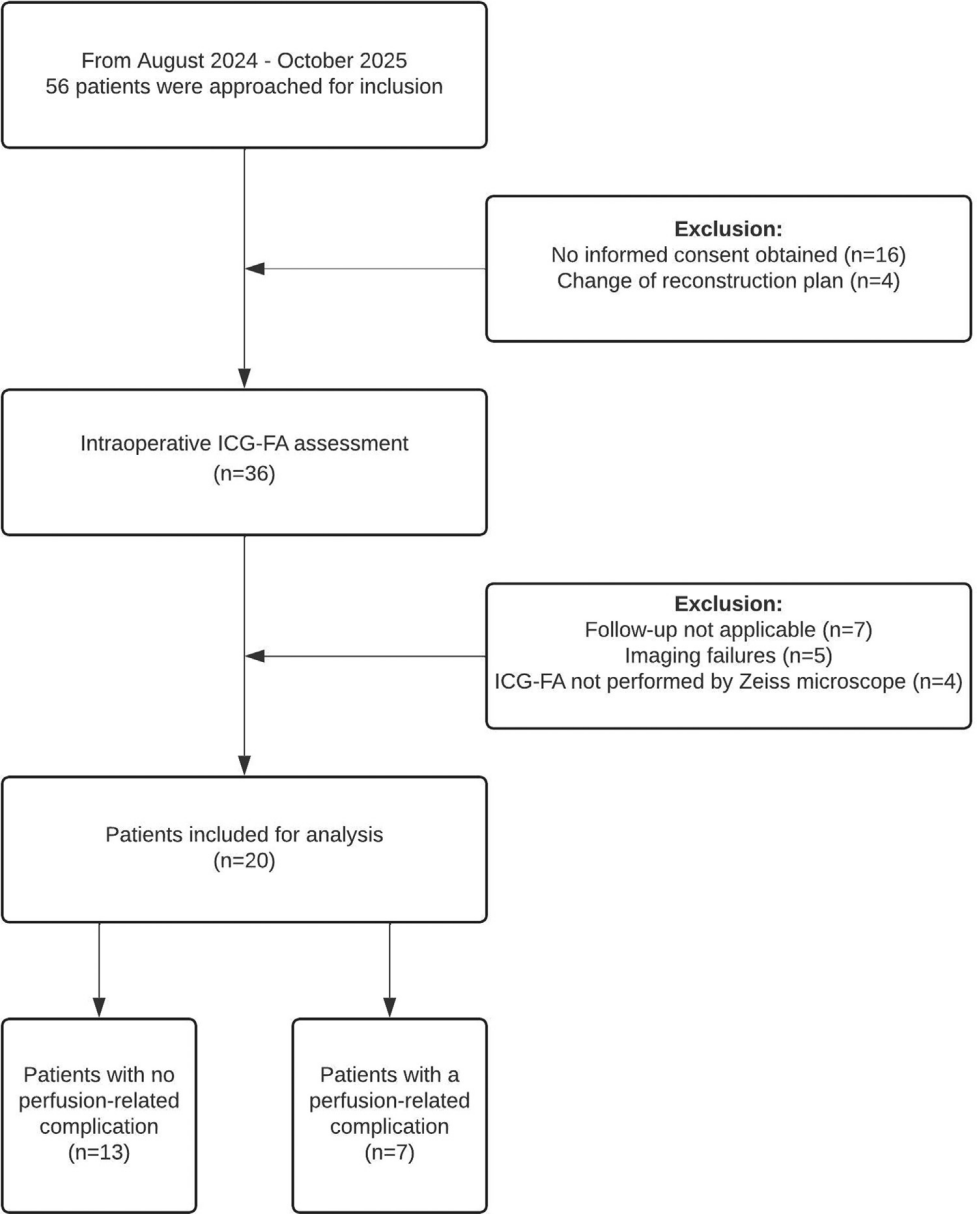
Flowchart of included patients. Follow up not applicable: buried flap which was invisible for postoperative inspection (*n* = 5), flap revision directly after ICG-FA (*n* = 2). Imaging failures: light intensity failures (*n* = 3), distal flap area outside area of interest (*n* = 1), camera distance >60 cm (*n* = 1).

Between August 2023 and October 2024, 56 patients scheduled for fasciocutaneous flap reconstruction were screened for inclusion. Of these, 36 consented and underwent intraoperative ICG-FA. Sixteen were excluded for various reasons (Figure 3), resulting in a final cohort of 20 patients. In this cohort, 11 of the patients were male, with a mean age of 55 years (±15.5 years). No significant differences were observed between patients with and without perfusion-related complications in terms of comorbidities, including cardiovascular disease, diabetes, and renal disease. Baseline demographics, clinical characteristics and intraoperative hemodynamic parameters are presented in Table 1.

**Table 1:** Baseline demographics and clinical characteristics.

	Total *N* = 20	No PRC *N* = 13	PRC *N* = 7	*P*-value
Sex, male (%)	11 (55)	5 (38)	6 (86)	0.070
Age at time of surgery, mean ± SD (years)	55 ± 15.5	56 ± 15.2	53 ± 16.9	0.683
BMI, mean ± SD (kg/m^2^)	27.2 ± 4.0	26.6 ± 4.6	28.3 ± 2.8	0.379
ASA classification (ASA ≥ 3)	2 (10)	1 (8)	1 (14)	1.000
History of smoking, n (%)	10 (50)	5 (38)	5 (71)	0.350
Current smoker, n (%)	7 (23)	4 (31)	3 (43)	1.000
Reconstruction site, n (%)				
Lower extremity	6 (30)	2 (15)	4 (57)	
Thorax	5 (25)	5 (38)	0 (0)	
Perineal	3 (15)	2 (15)	1 (14)	
Head and neck	2 (10)	2 (15)	0 (0)	
Gluteal	2 (10)	1 (8)	1 (14)	
Upper extremity	1 (5)	0 (0)	1 (14)	
Abdominal	1 (5)	1 (8)	0 (0)	0.173
Flap type, n (%)				
Pedicled	16 (80)	11 (79)	5 (83)	
Free	4 (20)	3 (21)	1 (17)	1.000

PRC, perfusion-related complication; BMI, body mass index; ASA, American Society of Anesthesiologists Physical Status.

### Quantitative ICG-FA analysis

#### Fluorescence-time curves

**Figure 4:**
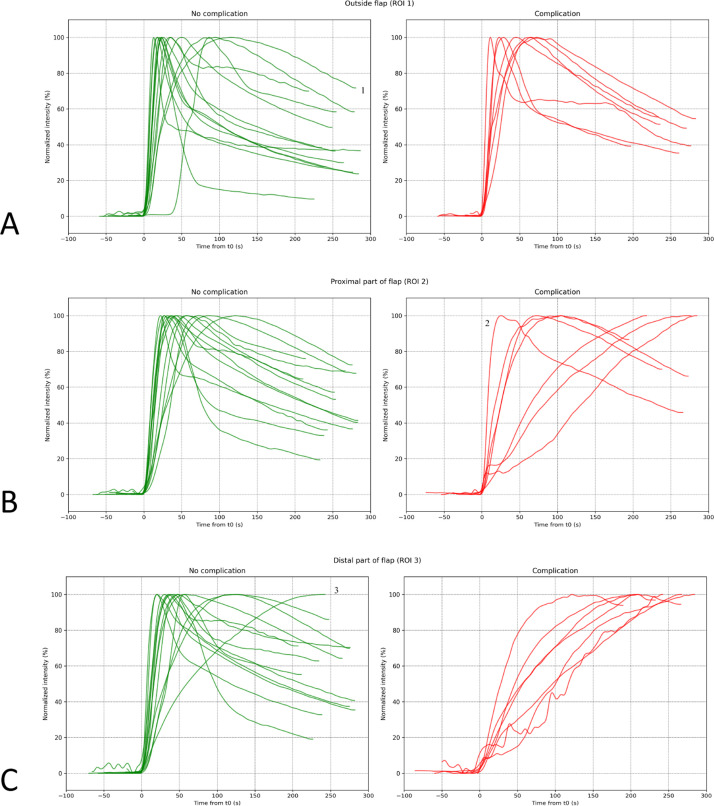
Comparison of normalized fluorescence-time-curves (FTC) across different regions of interest between patients with a perfusion-related complication (red) and patients without (green). (a) ROI1 shows similar FTC patterns between the No PRC and PRC groups, indicating normal tissue perfusion. ^1^FTC corresponds to a patient who underwent a DIEP reconstruction and had clinically malperfused native skin (reference area). (b) In ROI2, the No PRC group has consistent FTC patterns, while the PRC group exhibits variability, including slower ingress and reduced or absence egress. ^2^FTC represents a patient who did not develop complications in ROI2 but experienced distal necrosis in ROI3. (c) In ROI3, the No PRC group generally shows rapid ingress and variable egress, whereas the PRC group demonstrates prolonged ingress with minimal egress. ^3^FTC corresponds to a patient without a PRC; however, the FTC shows decreased ingress and no outflow.

Normalized FTCs for all three ROIs in the No PRC and PRC groups are shown in Figure 4. In the PRC group, most FTCs showed a delayed increase in fluorescence intensity and minimal or absent washout, particularly in the distal region (ROI3). These patterns typically resulted in broader, flatter curves with a delayed fluorescence peak. In the No PRC group, FTCs generally demonstrated rapid inflow, a well-defined peak, and gradual washout. FTC characteristics showed variability between individual patients, but overall trends differed clearly between the PRC and No PRC groups, consistent with the quantitative differences reported below.

#### Quantitative parameters

Q-ICG-FA parameters were compared between the No PRC and PRC group (Table 2). As for the ingress parameters, a significant delay in the *Ttp* was observed in the ‘PRC’ group compared to the No PRC group within the flap tissue in ROI2 (median of 105 s vs. 36, *p* = 0.013) and even more so in ROI3 (median of 209 s vs. 48, *p* = 0.001). The PRC group demonstrated a significantly lower *normalized* *mean* *slope* *inflow* within the flap tissue compared to the No PRC group, in both ROI2 (median of 0.9%/s vs. 2.4%/s, *p* = 0.029) and in ROI3 (median of 0.5%/s vs. 2.1%/s, *p* = 0.001). The PRC group exhibited a significant lower *normalized* *maximum* *slope* *inflow* within the flap tissue, specifically in ROI3 (median of 1.6%/s vs. 4.2%/s, *p* = 0.002). As for the egress parameters, a significant reduction in the *normalized* *mean* *slope* *outflow* was observed in the PRC group in ROI3 (median of 0.1%/s vs. 0.2%/s, *p* = 0.021).

**Table 2:** Quantitative ICG-FA parameters.

Parameter	ROI1 Normal vascularized reference tissue			ROI2 Proximal flap tissue			ROI3 Distal flap tissue		
	No PRC *N* = 13	PRC *N* = 7	*p*	No PRC *N* = 13	PRC *N* = 7	*p*	No PRC *N* = 13	PRC *N* = 7	*p*
Ingress									
*Ttp* (*sec*)	25 (13–67)	45 (11–72)	0.322	36 (22–123)	105 (25–287)	**0.024**	48 (20–241)	209 (122–291)	**0.001**
*Fmax (AU)*	105 (7–223)	39 (14–176)	0.191	81 (11–230)	46 (14–115)	0.122	72 (11–184)	28 (8–95)	0.075
Norm mean slope (%/s)	3.4 (1.1–6.8)	2.1 (1.3–6.8)	0.322	2.4 (0.8–3.7)	0.9 (0.3–3.5)	**0.029**	2.1 (0.4–3.9)	0.5 (0.3–0.8)	**0.001**
Norm max slope (%/s)	6.2 (3.8–12.6)	5.6 (3.3–14.0)	0.663	5.2 (2.0–7.4)	2.6 (1.0–8.9)	0.052	4.2 (1.2–10.0)	1.6 (1.0–2.9)	**0.002**
Egress									
Norm mean slope (%/s)	0.3 (0.3–0.4)	0.3 (0.2–0.4)	0.428	0.2 (0.1–0.4)	0.2 (0.1–0.2)	0.258	0.2 (0.1–0.4)	0.1 (0.1–0.1)	**0.021**
Norm max slope (%/s)	1.3 (0.3–4.2)	0.6 (0.4–2.6)	0.452	0.6 (0.2–2.1)	0.3 (0.3–1.0)	0.213	0.8 (0.2–1.4)	0.3 (0.2–0.3)	**0**.112

Values are in median (range). PCR, perfusion-related complication. ROI, region of interest. No PRC, patients without a perfusion-related complication.

PRC, patients with a perfusion-related complication; T_0_, the moment at which the fluorescence intensity within the ROI exhibited a statistically significant increase over the background level; Ttp, time from t0 to maximum fluorescence intensity, F_max_, the maximum fluorescence intensity.

### Clinical outcomes

Perfusion-related complications occurred in seven patients, with venous congestion being the most common (*n* = 5). Revision surgery was performed in three patients. Partial flap failure occurred in two patients, and complete flap failure in one. An overview of flap characteristics, type of complication, management, and outcomes is provided in Table 3. Flap types and vascular patterns were distributed across both groups without an apparent relationship to the occurrence of perfusion-related complications; no subgroup analysis was performed due to the small sample size.

**Table 3:** Flap characteristics and clinical outcomes.

Case	Flap type	Transfer type	Vascularization	Reconstruction site	Intraoperative clinical assessment	PRC	Flap outcome
1	Transposition	Pedicled	Perforator (PTA)	Lower extremity	Viable	No	Viable
2	Parascapular	Free	Axial	Head & neck	Viable	No	Viable
3	Forehead flap	Pedicled	Axial	Head & neck	Viable	No	Viable
4	Propeller flap	Pedicled	Perforator (PTA)	Lower extremity	Viable	Venous congestion	Salvaged after venous revision
5	SCIP	Pedicled	Perforator	Abdomen	Viable	No	Viable
6	V-Y advancement	Pedicled	Perforator (PTA)	Lower extremity	Viable	No	Viable
7	Lotus petal flap	Pedicled	Perforator	Perineal	Viable	No	Viable
8	DIEP	Free	Perforator	Thorax	Viable	No	Viable
9	Gluteal transposition	Pedicled	Random	Gluteal	Viable	Wound dehiscence due to suboptimal perfusion	Viable, secondary reconstruction planned
10	Lotus petal V-Y flap	Pedicled	Perforator	Perineal	Congested, managed with gauzes soaked in heparin	Venous congestion, wound dehiscence	Salvaged after controlled bleeding. Wound dehiscence was managed with vacuum therapy
11	PIA flap	Pedicled	Axial	Upper extremity	Viable	Venous congestion	Salvaged after pressure release
12	DIEP	Free	Perforator	Thorax	Viable	No	Viable
13	ALT flap	Free	Perforator	Lower extremity	Viable	Venous congestion with distal flap necrosis	Salvaged after venous revision with eventual partial flap failure
14	Gluteal transposition	Pedicled	Random	Gluteal	Viable	No	Viable
15	TDAP flap	Pedicled	Perforator	Thorax	Viable	No	Viable
16	Parascapular	Free	Axial	Lower extremity	Viable	Venous congestion	Revision failed; complete flap failure
17	Ponten	Pedicled	Random	Lower extremity	Viable	Distal necrosis	Partial flap failure
18	V-Y advancement	Pedicled	Random	Perineal	Viable	No	Viable
19	Rhomboid flap	Pedicled	Random	Thorax	Viable	No	Viable
20	DIEP	Free	Perforator	Thorax	Viable	No	Viable

PTA, posterior tibial artery; DIEP, deep inferior epigastric perforator; SCIP, superficial circumflex iliac perforator; ALT, anterolateral thigh; TDAP, thoracodorsal artery perforator; PIA, posterior interosseous artery flap.

## Discussion

This pilot study demonstrates that Q-ICG-FA can effectively differentiate between fasciocutaneous flaps with perfusion-related complications and those that heal uneventfully. These differences were characterized by broader FTCs with a delayed peak and slower washout in the compromised flaps, compared to the more rapid increase and a well-defined peak observed in the uncomplicated flaps. These findings suggest that Q-ICG-FA can objectively detect perfusion disturbances that may not be recognized through conventional intraoperative assessment.

ICG-FA has become an important adjunct in reconstructive surgery, with increasing interest in quantitative analysis. As summarized by Andersen et al.,^23^ Q-ICG-FA parameters can be categorized into intensity-based, time-related, slope-based, and relative indices. Despite the extensive variety of quantitative parameters described, most clinical applications still rely on relative intensity thresholds, such as the widely used 33% cut-off for mastectomy skin flaps,^32^, ^33^, ^34^, ^35^, ^36^ which most likely represents ischemia. However, the applicability of this threshold to other flap types, particularly those with venous outflow issues, remains uncertain. In this study, venous congestion was the most common complication, exhibiting a characteristic FTC pattern. These cases showed a delayed or diminished inflow rate, followed by plateauing and poor or absent outflow. This pattern likely reflects elevated intra-flap pressure hindering venous drainage, leading to fluorescence stagnation. Similar findings have been reported in experimental comparisons of arterial and venous occlusion, including prior work from our group.^37^ Relative intensity thresholds fail to detect these perfusion abnormalities, as they measure overall fluorescence intensity, which primarily reflects the amount of dye present but does not capture the dynamic nature of blood flow. This limitation becomes particularly evident in cases of venous congestion, where slow inflow and fluorescence stagnation may occur without significant changes in overall fluorescence intensity. As such, relative intensity thresholds are less effective at identifying these disturbances, which can lead to missed diagnoses. The findings of the present study are of clinical relevance, as they demonstrate the ability of Q-ICG-FA to identify venous outflow issues intraoperatively. This potential has been largely underexplored in the literature, despite the role of venous congestion as a frequent cause of flap failure.^7^^,^^38^

Exploratory observations suggested potential differences in FTC morphology between flap types and vascular patterns; for example, pedicled flaps appeared to show more pronounced distal delayed perfusion as compared to free flaps. However, the small number of cases precludes meaningful comparison, and these findings should be interpreted cautiously. Previous experimental work has demonstrated differences between perforator-based and random flaps, supporting the possibility that flap-specific perfusion characteristics influence FTC behavior.^39^ Larger, stratified studies will be required to investigate these preliminary observations and to clarify how flap type and vascular pattern shape FTC morphology.

The variability observed between individual FTCs highlights the complexity of defining universal perfusion thresholds. While establishing quantitative cut-off values remains an important goal, qualitative interpretation of curve morphology may offer additional clinical insight. Intraoperatively, rapid recognition of decreased inflow, plateauing, or absent washout may alert surgeons to potential perfusion compromise. Nevertheless, ICG-FA represents a single time-point assessment. A flap displaying an atypical distal curve may still recover through vascular adaptation, while a flap with normal intraoperative perfusion may subsequently fail due to acute postoperative events such as thrombosis.^40^^,^^41^ These nuances underline the need for cautious interpretation and complementary postoperative monitoring.

### Limitations

This pilot study has several limitations. The sample size was small, limiting statistical power and precluding reliable subgroup analyses by flap type or vascular pattern. Heterogeneity in flap type and patient comorbidities may influence perfusion dynamics and therefore represents a potential source of confounding. Specific intraoperative details, such as flap dimensions, were not systematically recorded. Although perfusion-related complications were defined a priori, their classification depended on clinical documentation and part of the dataset was retrospectively extracted from clinical records.

The microscope-integrated ICG-FA system offered consistent imaging conditions but a limited field of view, reducing assessment of global flap perfusion compared with wide-field systems such as QUEST. Finally, normalization of FTCs facilitated comparison between cases but may mask clinically relevant differences in absolute fluorescence, particularly in severely hypoperfused tissue.^30^

### Future directions

Future studies should include larger cohorts to validate these findings and further explore perfusion dynamics across flap types and vascular patterns. The characteristic FTC profile observed in venous congestion, would be very interesting to explore in more detail. Incorporating postoperative ICG-FA could clarify how intraoperative perfusion patterns relate to postoperative perfusion status and clinical outcomes. Although microscope-integrated systems offer seamless workflow integration, their relatively small field of view limits assessment of entire flaps, and comparison with established wide-field systems such as QUEST will be important.

## Conclusion

This pilot study demonstrates that Q-ICG-FA can objectively distinguish fasciocutaneous flaps with perfusion-related complications from those with uncomplicated healing. Distinct FTC features, particularly decreased inflow and impaired washout, were associated with compromised perfusion, most notably in cases of venous congestion. These findings support the potential role of Q-ICG-FA as an intraoperative adjunct for early detection of perfusion disturbances. Larger prospective studies are needed to validate these observations and refine the clinical application of Q-ICG-FA in reconstructive surgery.

## Declaration of Competing Interest

MvBH is a consultant for Intuitive, Medtronic, Johnson & Johnson, Stryker, and B. Braun (all fees paid to the institution). RH has received payments for lectures from Stryker. The other authors declare no conflicts of interest.
